# The complete mitochondrial genome of *Xanthomantis bimaculata* (Mantodea: Iridopterygidae) and its phylogeny

**DOI:** 10.1080/23802359.2020.1797593

**Published:** 2020-07-30

**Authors:** Jia-Yin Guan, Yi-Yang Jia, Zi-Yi Zhang, Si-Si Cao, Jin-Liang Ma, Jia-Yong Zhang, Dan-Na Yu

**Affiliations:** a College of Chemistry and Life Science, Zhejiang Normal University, Jinhua, China; b Key Lab of Wildlife Biotechnology, Conservation and Utilization of Zhejiang Province, Zhejiang Normal University, Jinhua, China

**Keywords:** *Xanthomantis bimaculata*, Iridopterygidae, mitogenome, phylogenetic relationship

## Abstract

The mitochondrial genome sequence of *Xanthomantis bimaculata* (Mantodea: Iridopterygidae) from Yunnan, China is a circular molecule with the typical insect mitochondrial gene arrangement, which is 15,941 bp in length and contains 22 tRNAs, two rRNAs, 13 protein-coding genes, and one control region. The overall AT content of the mitogenome is 73.12% (A = 37.58%, T = 35.54%, C = 16.54%, G = 10.34%). In BI and ML phylogenetic analyses, *X. bimaculata* was a sister clade to *Sceptuchus simpl*ex. The monophyly of the families Iridopterygidae, Thespidae and Liturgusidae were supported.

Mantises are one of the most widely recognized groups of insects, which are divided into 15 families with 2400 species of 430 genera (Wang et al. 2016). Species of the Iridopterygidae family are small, slender group of mantids. Species of Iridopterygidae distributed in tropical Africa, southern Asia, and Australia are divided into five subfamilies (Svenson and Whiting 2009). More and more mitochondrial genomes of mantis were used to discuss the relationship within Mantodea (e.g. Zhang et al. 2018a, 2018b; Jia et al. 2019; Zhang et al. 2019). Obtaining more complete mitochondrial genome sequences of Mantodea will be beneficial to the further study of the phylogenetic relationship of mantis. Therefore, in this study, the complete mitochondrial genome of *Xanthomantis bimaculata* (Mantodea: Iridopterygidae) was sequenced.

The sample of *X. bimaculata* (2015XSBN0707) was collected from Xishuangbanna (N 101.257°, E 21.9242°), Yunnan Province, China. The sample was identified and stored at −40 °C in the Animal Specimen Museum, College of Life Sciences and Chemistry, Zhejiang Normal University, China. Total genomic DNA was extracted from one leg tissue using Ezup Column Animal Genomic DNA Purification Kit (Sangon Biotech Company, Shanghai, China) and stored in the Dr. JY Zhang’s laboratory. A set of modified universal primers (Zhang et al. 2008; Zhang et al. 2018) was designed for polymerase chain reaction (PCR) amplification. Subsequently, the remaining gaps were sequenced by utilizing species-specific primers according to previously obtained sequences. All PCR products were sequenced in both directions by the Sangon Biotech Company (Shanghai, China). The mitochondrial genome was deposited in GenBank with an accession number MT679725.

The mitogenome of *X. bimaculata* is a circular DNA molecule with a total length of 15,941 bp. The overall AT content of the whole mitogenome is 73.12% (A = 37.58%, T = 35.54%, C = 16.54%, G = 10.34%). In the 13 protein-coding genes, *COXI* gene uses TTG as the start codon, whereas the remaining 12 protein-coding genes use ATN (N stands for A, T, C, G) as the start codon. Most protein-coding genes use TAA as a stop codon. However, *ND3* uses ATG as a stop codon, and *COXI*, *COXII*, *COXIII*, and *ND5* end with an incomplete stop codon (T––).

In order to construct a phylogenetic relationship of *X. bimaculata*, 38 mitochondrial genome sequences of Mantodea (Cameron et al. 2006; Ye et al. 2016; Tian et al. 2017; Zhang and Ye 2017; Zhang et al. 2018) were downloaded from GenBank and four mitochondrial genome sequences of Blattodea species (Zhang et al. 2010; Wei et al. 2012; Dietrich and Brune 2016) were selected as outgroups. Phylogenetic relationships were reconstructed using the Bayesian inference (BI) method implemented in MrBayes version 3.1.2 (Huelsenbeck and Ronquist 2001) and maximum-likelihood (ML) in RAxML 8.2.0 (Stamatakis 2014) based on 13 PCGs. To select conserved regions of the putative nucleotide sequences, each alignment was analyzed with Gblocks 0.91b (Castresana 2000) using default settings. In BI and ML phylogenetic trees, *X. bimaculata* was a sister clade to *Sceptuchus simplex.* The monophyly of the families Iridopterygidae, Thespidae and Liturgusidae were supported both in BI and ML analyses (Figure 1).

**Figure 1.**
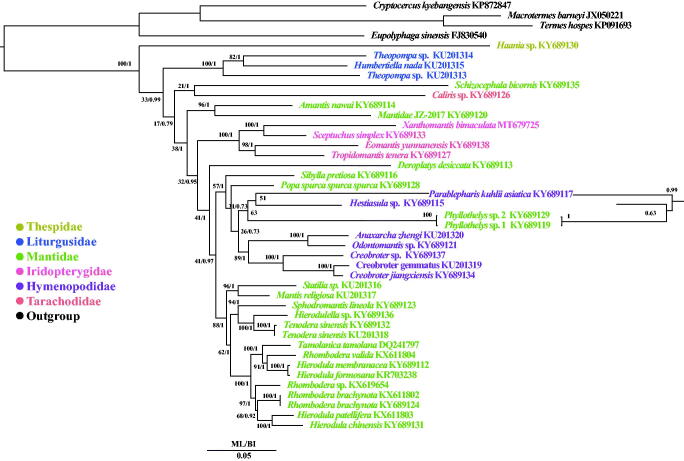
Phylogenetic tree of the relationships among 39 species of Mantodea including *Xanthomantis bimaculata* and four species of Blattodea, were based on the nucleotide dataset of the 13 mitochondrial protein-coding genes. Numbers around the nodes are the posterior probabilities of BI (right) and the bootstrap values of ML (left). The GenBank numbers of all species are shown in the figure.

## Data Availability

The data that support the findings of this study are openly available in NCBI at www.ncbi.nlm.nih.gov reference number [MT679725]. The alignment data are openly available in ZJNU data at https://cloud.zjnu.edu.cn/share/b8f44b9225f2d839aca9b2a44c.

## References

[CIT0001] Cameron SL, Barker SC, Whiting MF. 2006. Mitochondrial genomics and the new insect order Mantophasmatodea. Mol Phylogenet Evol. 38(1):274–279. 16321547 10.1016/j.ympev.2005.09.020

[CIT0002] Castresana J. 2000. Selection of conserved blocks from multiple alignments for their use in phylogenetic analysis. Mol Biol Evol. 17(4):540–552. 10742046 10.1093/oxfordjournals.molbev.a026334

[CIT0003] Dietrich C, Brune A. 2016. The complete mitogenomes of six higher termite species reconstructed from metagenomic datasets (*Cornitermes* sp., *Cubitermes ugandensis, Microcerotermes parvus, Nasutitermes corniger, Neocapritermes taracua, and Termes hospes*). Mitochondrial DNA A DNA Mapp Seq Anal. 27(6):3903–3904. 25471441 10.3109/19401736.2014.987257

[CIT0004] Huelsenbeck JP, Ronquist F. 2001. MRBAYES: Bayesian inference of phylogenetic trees. Bioinformatics. 17(8):754–755. 11524383 10.1093/bioinformatics/17.8.754

[CIT0005] Jia YY, Zhang LP, Xu XD, Dai XY, Yu DN, Storey KB, Zhang JY. 2019. The complete mitochondrial genome of *Mantis religiosa* (Mantodea: mantidae) from Canada and its phylogeny. Mitochondrial DNA B. 4(2):3797–3799. 10.1080/23802359.2019.1681912 PMC7707471 33366196

[CIT0006] Stamatakis A. 2014. RAxML version 8: a tool for phylogenetic analysis and post-analysis of large phylogenies. Bioinformatics. 30(9):1312–1313. 24451623 10.1093/bioinformatics/btu033 PMC3998144

[CIT0007] Svenson GJ, Whiting MF. 2009. Reconstructing the origins of praying mantises (Dictyoptera, Mantodea): the roles of Gondwanan vicariance and morphological convergence. Cladistics. 25(5):468–514. 10.1111/j.1096-0031.2009.00263.x 34879623

[CIT0008] Tian XX, Liu J, Cui Y, Dong PZ, Zhu Y. 2017. Mitochondrial genome of one kind of giant Asian mantis, *Hierodula formosana* (Mantodea: Mantidae). Mitochondrial DNA A DNA Mapp Seq Anal. 28(1):11–12. 26641534 10.3109/19401736.2015.1106519

[CIT0009] Wang TT, Yu PP, Ma Y, Cheng HY, Zhang JY. 2016. The complete mitochondrial genome of *L. albella* (Mantodea: Iridopterygidae). Mitochondrial DNA A DNA Mapp Seq Anal. 27(1):465–466. 24660931 10.3109/19401736.2014.900669

[CIT0010] Wei SJ, Ni JF, Yu ML, Shi BC. 2012. The complete mitochondrial genome of *Macrotermes barneyi light* (Isoptera: Termitidae). Mitochondrial DNA. 23(6):426–428. 22920221 10.3109/19401736.2012.710215

[CIT0011] Ye F, Lan XE, Zhu WB, You P. 2016. Mitochondrial genomes of praying mantises (Dictyoptera, Mantodea): rearrangement, duplication, and reassignment of tRNA genes. Sci Rep. 6:25634. 27157299 10.1038/srep25634 PMC4860592

[CIT0012] Zhang LP, Cai YY, Yu DN, Storey KB, Zhang JY. 2018a. The complete mitochondrial genome of *Psychomantis borneensis* (Mantodea: Hymenopodidae). Mitochondrial DNA B. 3(1):42–43. 10.1080/23802359.2017.1419094 PMC7800046 33474058

[CIT0013] Zhang LP, Cai YY, Yu DN, Storey KB, Zhang JY. 2018b. Gene characteristics of the complete mitochondrial genomes of *Paratoxodera polyacantha* and *Toxodera hauseri* (Mantodea: Toxoderidae). PeerJ. 6:e4595. 29686943 10.7717/peerj.4595 PMC5911385

[CIT0014] Zhang LP, Ma Y, Yu DN, Storey KB, Zhang JY. 2019. The mitochondrial genomes of *Statilia maculata* and *S. nemoralis* (Mantidae: Mantinae) with different duplications of trnR genes. Int J Biol Macromol. 121:839–845. 30340009 10.1016/j.ijbiomac.2018.10.038

[CIT0015] Zhang YY, Xuan WJ, Zhao JL, Zhu CD, Jiang GF. 2010. The complete mitochondrial genome of the cockroach *Eupolyphaga sinensis* (Blattaria: Polyphagidae) and the phylogenetic relationships within the Dictyoptera. Mol Biol Rep. 37(7):3509–3516. 20012368 10.1007/s11033-009-9944-1

[CIT0016] Zhang HL, Ye F. 2017. Comparative mitogenomic analyses of praying mantises (Dictyoptera, Mantodea): origin and evolution of unusual intergenic gaps. Int J Biol Sci. 13(3):367–382. 28367101 10.7150/ijbs.17035 PMC5370444

[CIT0017] Zhang LP, Yu DN, Storey KB, Cheng HY, Zhang JY. 2018. Higher tRNA gene duplication in mitogenomes of praying mantises (Dictyoptera, Mantodea) and the phylogeny within Mantodea. Int J Biol Macromol. 111:787–795. 29307803 10.1016/j.ijbiomac.2018.01.016

[CIT0018] Zhang JY, Zhou CF, Gai YH, Song DX, Zhou KY. 2008. The complete mitochondrial genome of *Parafronurus youi* (Insecta: Ephemeroptera) and phylogenetic position of the Ephemeroptera. Gene. 424(1-2):18–24. 18725275 10.1016/j.gene.2008.07.037

